# Child-to-Parent Violence Specialist and Generalist Perpetrators: Risk Profile and Gender Differences

**DOI:** 10.3390/healthcare11101458

**Published:** 2023-05-17

**Authors:** Ismael Loinaz, Maialen Irureta, César San Juan

**Affiliations:** 1 Department of Clinical Psychology and Psychobiology, University of Barcelona, 08035 Barcelona, Spain; 2 Department of Social Psychology, University of the Basque Country UPV/EHU, 20018 Donostia-San Sebastián, Spain; 3 Psychiatry Service, Donostia University Hospital, Begiristain Doktorea Pasealekua, 20014 Donostia-San Sebastián, Spain

**Keywords:** child-to-parent violence, risk factors, specialists, generalist, typologies

## Abstract

Like other forms of domestic violence, child-to-parent violence (CPV) is a social and health-related problem. The identification of risk factors has preventive and therapeutic implications. This paper analyzes the risk profiles and gender differences of 206 CPV cases between 12 and 28 years of age (58% males) from clinical and judicial contexts in Spain, assessed using the Child to Parent Violence Risk (CPVR) Assessment tool. Two profiles were compared according to the extent of their violence: those using only CPV (specialist, 64.1%) and those also using other types of violence (generalist, 35.9%), as coded by professionals working with the cases. Generalist perpetrators had a significantly higher prevalence in terms of the bidirectionality of the violence (being victims at home), bullying victimization, empathy problems, anger management issues, attitudes justifying violence, antisocial behavior, failure of previous interventions, violence between parents, cohabitation problems other than CPV, problematic education style, and inversion of the hierarchy. Females were less likely to be generalists, and, in the case of female specialists, violence from parents and issues in the family context may have been among the main explanations for their violence. The results suggest differences between groups, which is consistent with previous research, but also the need for more accurate typological classification methods.

## 1. Introduction

Like other forms of domestic violence, child-to-parent violence (CPV) is a social and health-related problem with many facets and variables involved. CPV consists of repeated physical, psychological, or economic aggression directed at parents or those who play a parental tole [1]. Although it is almost exclusively associated with adolescents and minors, it can be perpetrated by aggressors of different ages, including adults. In fact, some recent studies have highlighted this situation [2], specifically describing the risk and protective factors among emerging adults (18–23 years) [3] or concluding that there is a CPV offender profile that is common in adolescents and adults [4]. In addition to the differing ages of the perpetrators, they can be almost equally male and female (depending on the sample), with similar risk factors between the genders (i.e., [3,5]); thus, there can be many different realities.

CPV figures are heterogeneous and depend on the sample (general population, clinical or judicial samples), the type of violence (psychological, physical, or economic; punctual or repeated), or the assessment instrument used [6]. Regarding specific assessment tools, several proposals have emerged in recent years that attempt to address the problem, such as self-reporting instruments designed to quantify or categorize the type of violence (the *Child-to-Mother Violence Scale* [7], the *Child-to-Parent Violence Questionnaire* (CPV-Q) [8], the *Child-to-Parent Aggression Questionnaire* (CPAQ) [9], and *Abusive Behavior by Children—Indices* (ABC-I) [10]); tools to classify perpetrators (the *Adolescent Domestic Battery Typology* [11]); a risk assessment tool (the *Child-to-Parent Violence Risk Assessment Tool* (CPVR) [12]); and the caregiver report measure to learn the function of CPV (the *Child-to-Parent Violence Functions Scale* (CPV-F) [13]).

Research on risk factors related to CPV has increased in recent years [14], describing personal variables, family context, and gender differences and similarities, among other factors [12,15,16]. It has also focused on reviewing specific variables, such as the presence of victimization by parents [17] and the influence of drugs [18]. Despite this increase, few studies have attempted to develop a typological classification of CPV perpetrators. Four typologies of CPV perpetrators have been proposed according to their coercion level and type of violence [19]: offensive, defensive, affective, and situational (conflictive parent–child relationship). The comparison of CPV perpetrators to other types of offenders has been another research topic, with a higher presence of psychopathology and social–cognitive difficulties described in CPV cases [20], but also similarities in impulsivity [20,21] and self-esteem [20,22,23] in both samples. Moulds [24], in a study classifying CPV perpetrators, concluded that CPV offenses in isolation are rare, and most CPV perpetrators commit other non-violent or violent crimes. It has also been found that CPV cases have a higher risk (according to the YLS/CMI) and a higher prevalence of recidivism (with more offenses committed during follow-up) than young people who commit other offenses [25].

Recently, research on CPV typologies has compared “generalists” and “specialists” as two groups of perpetrators. Some authors [26,27] have classified aggressors according to callous–unemotional traits and the type of aggression. Generalist aggressors would be high in callous, unemotional traits and would perpetrate violence outside the home; specialists would be low in callous–unemotional traits and only perpetrate violence against their parents. These scholars proposed a trait-based model in which those high in callous–unemotional traits may differ in information processing and use proactive or cold-blooded violence, whereas specialists may be low in callous–unemotional traits, with their aggression occurring in response to provocation or harsh parenting [26]. The authors also provided a list of risk factors that can be used to classify offenders as generalists (high callous–unemotional traits; proactive peer aggression; permissive parenting; domination motivation; and violence toward their father, mother, sibling, or peers) or specialists (low callous–unemotional traits, reactive peer aggression, harsh parenting, revenge motivation, and violence toward father or mother). Boxal [28] also concluded that not all CPV cases follow the same patterns of behavior, which is helpful in distinguishing between generalists and specialists. For example, those who target their mothers are unlikely to perpetrate violence against other family members or people outside the family, while other perpetrators may be violent against different people, with CPV being only part of their overall criminal career. The generalist profile has even been associated with insensitivity characteristics, more instrumental violence, and an early age of onset of CPV (10–11 years) [29].

Similarly, a research line on specialist versus generalist CPV perpetrators was conducted in the Spanish context. In a sample of 208 Spanish juvenile offenders (83 CPV and 126 other offenses), CPV perpetrators had more socio-cognitive problems, less parental warmth, more parental criticism/rejection, and higher prevalence of different forms of victimization (directly, at home, and indirectly, at school or on the street) [30]. The results suggested that CPV generalist perpetrators are older and have worse profiles than specialists, with more justification for violence, impulsivity, drug problems, parental criticism or rejection, exposure to violence on the street, and deviant peers. In another sample of 1559 Spanish CPV perpetrators from educational centers, 22.4% specialists and 77.6% generalists were described [31]. Again, generalists had worse profiles than specialists, with more reactive and proactive CPV, less emotional intelligence and resilience, more insecure parental attachment, and more violence from parents (direct or witnessed). In the same sample, no differences were described between females and males in the specialist group, but they were present in the generalist group [32]. Generalist girls used a higher level of psychological and controlling violence against their mothers, and males committed more physical violence against their fathers. Among specialists, the use of violence by parents predicted reactive CPV, whereas parental permissiveness was related to proactive CPV. Specialist CPV perpetrators used less CPV than generalists, with no differences between females and males. Among generalists, girls used subtle forms of CPV and boys more direct forms.

The aim of the current study was to compare different risk profiles of CPV perpetrators, according to the aforementioned classifications, using a recently developed CPV-specific risk assessment tool. This was carried out, first, to learn how generalist and specialist perpetrators differ in their risk and protective factors; and second, to analyze whether there are differences between genders in these subtypes of perpetrators. The hypotheses based on previous research were that: (1) two types of CPV perpetrators could be distinguished according to their use of violence [26,27,28,29]; (2) generalist perpetrators would have a worse risk profile than specialists [30,31]; and (3) some gender differences would emerge [3,32], mainly worse environments (specifically, family issues) for females [5].

## 2. Materials and Methods

### 2.1. Participants

The sample consisted of 206 subjects from different services in Spain: from clinical centers (Amalgama7, *n* = 49; Euskarri, *n* = 10; and Recurra-Ginso, *n* = 116), from the judicial system (*n* = 22), and from judicial measures in a foundation (Pioneros *n* = 9). Those from a judicial measure context were serving sentences in a therapeutic center, while those in the judicial system were assessed as requiring judicial advice prior to any intervention. Their mean age was 16.33 years (*SD* = 1.95; range = 12–28). There were 119 males (58%) and 87 females (42%), and 95.2% (*n* = 197) were of Spanish nationality, although 12.4% were born outside of Spain. In the full sample, 47.1% (97) perpetrated CPV alone (without other violence or offenses); 16.9% (35) engaged in only CPV, but had committed other offenses (not violent in that other violence was not coded in the tool); and 35.9% (74) committed CVP, other types of violence, and other offenses. The types of offenses coded in the tool were other violent crimes (10.7%), drug-related crimes (17.5), and crimes against property (17%). For the current research, subjects were classified according to the specificity of their violence, as we did not have precise labeling of the criminal/antisocial activity. The first two groups were then coded as specialists (CPV only) and the remaining subjects as generalists (CPV and other violence).

### 2.2. Instrument

The *Child-to-Parent Violence Risk* (CPVR) assessment tool was used to assess the sample. The CPVR is a violence risk assessment tool included among the recently developed specific tools for CPV, with psychometric properties available [19]. The core tool consists of 24 risk factors divided into four domains (type of violence, psychological characteristics of the perpetrator, social adaptation of the perpetrator, and family factors), and 6 protective factors. Each factor is coded as present (evidence that the factor is present in the case), partially present (some evidence related to the item), or absent (evidence that the factor is absent in the case) for the present and past (before the last year at the time of the assessment). There is also an initial section on the coding sheet with more than 20 possible variables, including personal and family characteristics, history of violence, and type of victims. The tool was designed in accordance with international standards on violence risk assessment tools [12] by reviewing the research and available tools, gathering feedback from professionals, and piloting the draft version. It currently has a structured professional judgement approach format with no specific cut-off scores, but research using scores has shown utility in discriminating between clinical and judicial cases (AUC = 0.830) and in predicting the presence of injuries toward mothers in general (AUC = 0.764) [33], specifically by male versus female perpetrators [5]. It has also been used in the therapeutic context [34], highlighting that the CPVR may be sensitive to therapeutic change, but does not prove a relationship between the level of risk and a favorable or unfavorable clinical prognosis after the therapeutic intervention.

### 2.3. Procedure

Cases were assessed according to the usual procedure of each center, by their own staff. Therefore, the participants were patients in the clinical context, serving a sentence or being assessed within the juvenile justice system. In a second step, as part of the current project, the CPVR was coded from the case files by different professionals, either psychologists from each center (for cases from Euskarri, Fundación Pioneros and Recurra-Ginso) or masters students in forensic psychology participating in the research project and trained to use the tool (cases from Amalgama7 and the judicial system). The cases in the judicial system were assessed in the context of juvenile criminal justice, following a complaint, by a team of professionals working in the field of technical advice to the judiciary. Cases in judicial measures were assessed by therapeutic staff in the location where they were serving a judicial sentence (i.e., compulsory treatment). The use of types of violence other than CPV, recorded by professionals, was used as a criterion for the present research.

### 2.4. Analysis of Data

The chi square statistic was used for the comparison of proportions of risk and protective factors between specialists and generalists (with odds ratios) and for gender comparison, and mean score comparisons were made with the *t* student test. The global numerical level of risk was calculated by transforming risk factor codifications of 2 (Yes), 1 (?), and 0 (No).

## 3. Results

### 3.1. Differences by Type of Violence

First, CPV perpetrators were compared according to the extent of their violence. Item 2 of the CPVR scale (“violence other than CPV”) during the last year prior to the assessment (current moment) was used. Those who had perpetrated only CPV were coded as specialists (64.1%; *n* = 132), and the rest of the sample (who had also committed another type of violence) were coded as generalists (35.9%; *n* = 74).

According to the sociodemographic profiles of the participants, both groups had roughly the same proportion of foreigners (5.4% generalists vs. 4.5% specialists) χ^2^ (1, *n* = 206) = 0.76, *p* = 0.783, and the same mean age at the time of the assessment (generalists, *M* = 16.06, *SD* = 1.60; specialists, *M* = 16.49, *SD* = 2.12) *t*(204) = 5.81, *p* = <0.001, but there were more males among the generalists (67.6% vs. 52.6%) χ^2^ (1, *n* = 206) = 4.299, *p* = 0.038. The age of onset of the violence was also the same in both groups (generalists, *M* = 12.02, *SD* = 3.33; specialists, *M* = 12.74, *SD* = 2.65) *t*(204) = −1.45, *p* = <0.111. The presence of other types of non-violent criminal activity was higher among generalists (48.6% vs. 26.5%) χ^2^ (1, *n* = 206) = 10.28, *p* = 0.001. Regarding the type of CPV, both groups committed the same type of violence against mothers and fathers, with no significant differences. There were no significant differences in the prevalences of generalist and specialist perpetrators coming from the clinical and judicial contexts (see Table 1).

Table 2 compares the prevalence of risk and protective factors of CPVR (the answers “yes” and “partially present” were combined for ease of comparison; see tools section). Prevalence was higher among generalist perpetrators, except for psychopathological symptomatology. Prevalence was significantly higher among generalists for bidirectionality of the violence (being victimized at home), bullying victimization, empathy problems, anger management issues, attitudes or beliefs justifying violence, antisocial behavior, failure in previous interventions, violence between parents, cohabitation problems other than CPV, problematic education style, and inversion of the hierarchy. Regarding the CPVR dimensions, most differences increasing the risk of being a generalist appeared in family factors (OR 2.09–2.67). There were also differences in protective factors (higher prevalence among specialists), which reduce the likelihood of committing a generalist violence, namely, the presence of future plans, social support, and family support (OR = 0.26–0.45).

Regarding the overall level of risk (score on the 24 CPVR risk factors), the scores were significantly higher among generalist perpetrators (*M* = 28.56, *SD* = 7.09 vs. *M* = 21.31, *SD* = 9.24), *t*(204) = 5.81, *p* = <0.001, indicating a global risk profile that is worse among generalists.

### 3.2. Gender Differences by Type of Violence

Gender differences between both types of perpetrators are shown in Table 3. Among specialists, females suffered significantly more violence from their parents and witnessed more violence between them. They had more complaints, more self-esteem problems, more cohabitation problems, and their parents had more problems (i.e., drug use or mental disorders). Among the generalists, females suffered from more bullying, cohabitation problems, and non-violent conflict between parents. Generalist females were also more motivated to change. Conversely, generalist males exhibited more empathy problems and academic difficulties. Males and females exhibited the same risk level among specialists (*M* = 20.50, *SD* = 9.23 vs *M* = 22.48, *SD* = 9.22), *t*(130) =1.212, *p* = 0.228, and among generalists, despite the higher scores of females (*M* = 27.97, *SD* = 7.15 vs *M* = 29.30, *SD* = 7.05), *t*(72) =.798, *p* = 0.427.

### 3.3. Intragender Differences between Specialist and Generalist

Intragender differences (Table 3 final columns) showed that, among male perpetrators, on the one hand, generalists experienced significantly more violence at home, CPV complaints, empathy problems, academic difficulties, antisocial behavior, failure of previous interventions, cohabitation problems other than CPV, and parents with personal problems. Specialists had significantly more motivation to change, future plans, and family support. Adaptation was the main area of difference between the two subtypes of perpetrators. Generalist females exhibited significantly more bullying victimization, anger management problems, attitudes justifying violence, antisocial behavior, cohabitation problems, and non-violent conflicts between parents compared to specialist females. Specialist females had significantly more future plans and social support.

## 4. Discussion

The existence of different types of CPV perpetrators has been a subject of research interest in recent years. Various hypotheses suggest the existence of emotional differences (such as the callousness–unemotional trait) [26], differences in victimization from parents [17,30,35], or differences in offending patterns among others [24]. It has been proposed that these differences are related to differences in the perpetration of CPV; for example, the use of proactive or reactive violence or the use of specific forms of violence against specific parents. The aim of the current research was to add more knowledge to these profiles using a sample of 206 CPV perpetrators coming from the justice system and from clinical/therapeutic contexts.

In the current sample, there were more specialists (those who only committed CPV, 64.1%) than generalists (who also committed another type of violence, 35.9%). These figures reflect previous research on juvenile justice services in the Spanish context [30], which found 68% specialists, but point in the opposite direction of the 77% of generalists that has been described among students [32]. Further, unlike Moulds [24], CPV offenses in isolation were not rare in the sample. It is worth noting that 47.1% only committed CVP (without other violence or offenses); 16.9% only committed CPV, but had other offenses or antisocial behaviors; and 35.9% committed CVP, other types of violence, and other offenses.

These discrepancies in the compositions of the groups confirm that there can be many differences between samples, depending on whether they are justice samples [24,30], mixed justice and clinical samples (as is the case in this study), or samples coming from the general population (i.e., students) [30,31,32]. The influence of the type of sample on the results has already been highlighted [6], and should always be taken into account in order to correctly analyze the results and statistics, as well as to avoid generalizing all CPV cases. Other influences may be the country or culture in which the research is developed [36] and also the specific type of CPV being assessed, such as physical or verbal CPV [37,38]. Some differences may be explained due to different criteria for labeling the cases as specialists or generalists, so all these questions should be addressed in the future.

When comparing these two types of perpetrators, the global prevalence of risk factors was higher among generalists. Bidirectionality of violence (being victims at home), bullying victimization, empathy problems, anger management issues, attitudes justifying violence, antisocial behavior, failure of previous interventions, violence between parents, cohabitation problems other than CPV, problematic education styles, and inversion of the hierarchy were significantly higher among them. The protective factors of future plans, social support, and family support were also associated with a reduced risk of being a generalist CVP perpetrator. Therefore, the results suggest some influence or application of protective factors, although the literature raises some doubts about the usefulness of these factors in juvenile offenders [39,40].

As is consistent with previous research, generalist perpetrators had higher levels of justification of violence [30]. They also had a higher prevalence of empathy problems, which is partly related to the callous–unemotional characteristics described by Kuay [26,27], and characteristics of perpetrators that interact with parental discipline [41]. In contrast to previous research [30], generalists and specialists did not differ in terms of impulsivity, drug use, age, or antisocial peers, and generalists experienced higher levels of victimization (at home and in school). For both groups, the same types of violence at home (physical, psychological, economic) were also used, and the same prevalence was found, contrary to what was found in previous research [31,32]. Both groups had the same age of onset of violence, so we could not confirm Curtis’ [29] hypothesis of an early onset in the generalist profile.

Broadly speaking, the main dimension differentiating the generalist group was family characteristics, such as violence between parents, conflict and problems, and even parents’ criminal history. This profile was consistent with cases from the judicial system in previous research comparing judicial and clinical cases [33]. In the case of female offenders females came from significantly more problematic contexts compared to males [5]. This could explain why and how these young people become violent in different contexts and begin criminal careers (social learning, disadvantaged and violent environments, criminal habits). It could also explain why females are almost as likely as males to be violent within the family although they are an absolute minority in all violent crimes.

In terms of gender, there were significantly more males among the generalist CPV perpetrators (67.6% vs. 52.6%), a gender distribution very similar to previous research on the judicial system [30] and in line with other works [24], but exactly the opposite of a study conducted on students by Navas-Martínez and Cano [32]. This again reflects the influence of the origin of the sample on the results. When comparing differences between male and female perpetrators, few differences emerge, as has been the case in other works [5,16]. Specialist females tended to suffer more violence at home and to have problematic family context, with intimate partner violence between parents, cohabitation problems (other than CPV), and personal problems in parents. This could explain their violence as a reaction to the context, a kind of reactive violence “against a hostile context”. Generalist females suffered more bullying and had more cohabitation problems and non-violent conflicts between parents. In other respects, they had the same risk profile as generalist males, so some females may also develop criminal careers motivated by the same causes.

There are several limitations to this study that must be considered to understand the results and implications. First, the type of violence (instrumental or reactive) was not assessed, so we could not go further in our interpretations of some results. Future research using the CPVR should include a tool such as the CPV-Q [8] to add this information. The variable used to classify perpetrators as generalists or specialists was only dichotomous, so it would be interesting to have a description of the specific types of violence perpetrated other than the CPV. All cases were CPV perpetrators, so a control/comparison group of non-offenders or other perpetrators who had not committed CPV would be of interest to assess the relevance of the described variables, or even the CPVR tool itself (to distinguish those who commit other types of violence or offenses from CPV perpetrators). This research focused on the differences according to the level of violence and gender differences between the two types of perpetrators, leaving aside the descriptions of profiles according to the origin of the sample (i.e., judicial or clinical), as has been the case in previous research [33]. This type of analysis could be of interest in the future, and may complement the results presented herein. Further, future research should pay attention to risk factors and different profiles according to age in order to deepen our knowledge of the different profiles of juvenile and adult perpetrators, as has been the case in recent research lines [2,3]. Finally, only bivariate analyses were conducted, so it could be of interest to improve CPV typological classifications using specific methods, such as cluster analysis.

This research adds to the literature on generalist and specialist CVP perpetrators, but some differences between studies may be related to research bias or methodological issues identified by Peck [42]. To improve research, we should consider the need to increase sample sizes, obtain representative samples, include outcome variables (such as recidivism), or use common definitions or categories of variables across studies. It is important to consider the limitations highlighted by Simmons et al. [14] in their review of 60 years of research on CPV; this is imperative to advancing our explanations about the origin, factors, and their combination in a more ecological way. To address gaps in our knowledge, there are some research questions which are partly related to the current paper (i.e., the relationship between CPV and broader antisocial behavior, or whether CPV can exist without a broader pattern of antisocial behavior) and should be investigated in future studies. Despite the findings of previous and current research on generalist and specialist CPV perpetrators, more research is needed on CPV typologies, as has been the case for other types of offenders, such partner-violent men (i.e., [43,44,45]) or sex offenders (i.e., [46,47]). The results may have practical implications for developing a better understanding the existing types of CPV offenders, establishing differences between forms and origins of violence, and thereby improving assessment and treatment systems.

## 5. Conclusions

In our sample, 64.1% of perpetrators did not use any other type of violence. The results confirmed the existence of at least two groups of CPV perpetrators—generalists and specialists—and some gender issues. Females were less likely to be generalists, and, in the cases of female specialists, violence by parents and the family context may have been among the main explanations for their violence. Discrepancies with previous studies highlighted the importance of differentiating the types of samples (which may partly explain the inconsistencies) and the need to develop more advanced typological classification systems, such as those developed for batterers or sexual offenders using cluster analyses. It is important to note that the definitions of specialists and generalists in this research were limited to the use of violence, so it will be necessary to contrast the results using different criteria (i.e., extent of violence, extent of antisocial activity, existence of criminal activity).

## Figures and Tables

**Table 1:** Differences in type of violence and other variables.

	Specialists (*n* = 132)		Generalists (*n* = 74)			
	*n*	%	*n*	%	χ^2^	*p*
**Academic/work situation**						
No work/no studies	12	9.5%	8	11.4%	0.734	0.865
studies	109	86.5%	60	85.7%		
work	1	0.8%	0	0%		
**Single-parent family**	53	40.5	29	39.7%	0.100	0.919
mother	48	90.6%	23	76.7%		
father	4	7.5%	5	16.5%		
aunt	1	1.9%	2	6.7%		
**Adoption**	14	10.8%	12	16.2%	1.258	0.262
**Immigration/family regrouping**	9	7.1%	6	8.6%	0.130	0.718
**Criminal history of parents**	11	9.5%	12	20.5%	4.234	0.040
**Violence toward mother**						
Physical	73	55.3%	51	68.9%	3.660	0.055
Psychological	126	95.5%	69	93.2%	0.459	0.498
Financial	73	55.3%	42	56.8%	0.041	0.840
Injuries	19	14.4%	17	23.0%	2.420	0.120
**Violence toward father**						
Physical	39	29.5%	27	36.5%	1.040	0.306
Psychological	80	60.6%	47	63.5%	0.170	0.681
Financial	43	32.6%	26	35.1%	0.139	0.709
Injuries	8	6.1%	3	4.1%	0.378	0.539
**Other criminal activity**	35	26.5%	36	48.6%	10.280	0.001
**Origin of the sample**						
Clinical	115	65.7%	60	34.3%	1.353	0.245
Judicial	17	54.8%	14	45.2%		

**Table 2:** Presence of risk factors in the current moment.

	Specialists (*n* = 132)		Generalists (*n* = 74)					C.I. 95%
	*n*	%	*n*	%	*χ* ^2^	*p*	OR	
**Risk factor (present)**								
**Violence**								
1. Bidirectionality	35	26.70%	30	40.50%	4.173	0.041	1.87	1.02–3.42
2. Violence other than CPV	-	-	-	-	-	-		
3. CPV complaints	16	12.20%	16	21.60%	3.177	0.075	1.98	0.92–4.24
4. Escalation	76	58.00%	51	68.90%	2.385	0.122	1.60	0.87–2,93
5. Bullying victimization	28	22.20%	25	35.20%	3.896	0.048	1.90	1.00–3.62
**Perpetrator Psychological Characteristics**								
6. Psychopathological symptomology	75	57.70%	40	54.10%	0.254	0.614	.863	0.49–1.53
7. Empathy problems	69	52.70%	55	74.30%	9.276	0.002	2.60	1.39–4.86
8. Self-esteem problems	97	74.60%	60	81.10%	1.112	0.292	1.46	0.72–2.95
9. Low frustration tolerance	108	81.80%	67	90.50%	2.822	0.093	2.13	0.87–5.21
10. Substance abuse	88	66.70%	52	70.30%	0.283	0.595	1.18	0.64–2.19
11. Impulsivity	104	78.80%	61	82.40%	0.395	0.530	1.26	0.61–2.62
12. Anger management issues	92	70.20%	66	89.20%	9.620	0.002	3.50	1.53–7.97
13. Narcissism and grandiose thoughts	31	23.80%	19	26.00%	0.120	0.729	1.12	0.58–2.18
14. Attitudes or beliefs justifying violence	46	35.70%	42	57.50%	9.074	0.003	2.45	1.36–4.40
**Adaptation**								
15. Academic difficulties	111	84.10%	66	89.20%	1.019	0.313	1.56	0.65–3.72
16. Antisocial behavior	66	50.40%	55	74.30%	11.208	0.001	2.85	1.53–5.32
17. Antisocial peers	78	59.10%	45	60.80%	0.058	0.809	1.07	0.60–1.92
18. Failure in previous interventions	90	70.90%	62	86.10%	5.920	0.015	2.55	1.18–5.50
**Family Factors**								
19. Violence between parents or guardians	24	18.30%	26	35.10%	7.250	0.007	2.41	1.26–4.63
20. Cohabitation problems other than CPV	52	39.40%	46	62.20%	9.856	0.002	2.53	1.41–4.54
21. Problematic education style	106	80.90%	68	91.90%	4.438	0.035	2.67	1.04–6.85
22. Inversion of the hierarchy	71	54.60%	53	71.60%	5.722	0.017	2.10	1.14–3.87
23. Personal problems of parents	40	30.30%	30	41.10%	2.435	0.119	1.61	0.88–2.91
24. Non-violent conflicts between parents	54	41.20%	44	59.50%	6.304	0.012	2.09	1.17–3.73
**Protective Factors**								
25. Motivation to change	73	57.00%	32	43.20%	3.571	0.059	0.57	0.32–1.02
26. Family involvement in therapy	105	80.20%	53	72.60%	1.530	0.216	0.66	0.34–1.28
27. Future plans	71	55.90%	18	25.00%	17.753	0.000	0.26	0.13–0.49
28. Social support	77	58.30%	28	37.80%	7.970	0.000	0.44	0.24–0.78
29. Family support	105	80.20%	47	64.40%	6.138	0.013	0.45	0.24–0.85
30. Working alliance in therapy	84	66.10%	39	52.70%	3.556	0.059	0.57	0.32–1.03

**Table 3:** Gender differences among specialists and generalists.

	Specialists						Generalists						Intragender			
	Male ^a^ (*n* = 78)		Female ^b^ (*n* = 54)				Male ^c^ (*n* = 41)		Female ^d^ (*n* = 33)				a vs. c		b vs. d	
	*n*	%	*n*	%	*χ* ^2^	*p*	*n*	%	*n*	%	*χ* ^2^	*p*	*χ* ^2^	*p*	*χ* ^2^	*p*
**Risk factor (present)**																
**Violence**																
1. Bidirectionality	14	18.2%	21	38.9%	6.951	0.008	16	39.0%	14	42.4%	0.088	0.767	6.130	0.013	0.106	0.744
2. Violence other than CPV	-	-	-	-	-	-	-	-	-	-						
3. CPV complaints	5	6.4%	11	20.8%	6.056	0.014	11	26.8%	5	15.2%	1.471	0.225	9.628	0.002	0.422	0.516
4. Escalation	47	61.0%	29	53.7%	0.701	0.402	31	75.6%	20	60.6%	1.921	0.166	2.535	0.111	0.397	0.529
5. Bullying victimization	14	19.2%	14	26.4%	0.930	0.335	9	22.5%	16	51.6%	6.489	0.011	0.176	0.675	5.409	0.020
**Perpetrator Psychological Characteristics**																
6. Psychopathological symptomology	46	60.5%	29	53.7%	0.602	0.438	25	61.0%	15	45.5%	1.774	0.183	0.002	0.962	0.558	0.455
7. Empathy problems	46	59.7%	23	42.6%	3.744	0.053	35	85.4%	20	60.6%	5.874	0.015	8.162	0.004	2.659	0.103
8. Self-esteem problems	52	67.5%	45	84.9%	5.002	0.025	31	75.6%	29	87.9%	1.794	0.180	0.837	0.360	0.150	0.699
9. Low frustration tolerance	64	82.1%	44	81.5%	0.007	0.933	38	92.7%	29	87.9%	0.493	0.483	2.481	0.115	0.621	0.431
10. Substance abuse	52	66.7%	36	66.7%	0	1	28	68.3%	24	72.7%	0.172	0.678	0.032	0.857	0.352	0.553
11. Impulsivity	59	75.6%	45	83.3%	1.130	0.288	34	82.9%	27	81.8%	0.016	0.901	0.835	0.361	0.033	0.856
12. Anger management issues	54	69.2%	38	71.7%	0.092	0.762	36	87.8%	30	90.9%	0.183	0.669	5.030	0.025	4.535	0.033
13. Narcissism and grandiose thoughts	19	24.4%	12	23.1%	0.028	0.867	13	32.5%	6	18.2%	1.925	0.165	0.887	0.346	0.290	0.590
14. Attitudes justifying violence	30	39.5%	16	30.2%	1.173	0.279	23	56.1%	19	59.4%	0.079	0.779	2.970	0.085	7.017	0.008
**Adaptation**																
15. Academic difficulties	65	83.3%	46	85.2%	0.082	0.775	40	97.6%	26	78.8%	6.683	0.010	5.241	0.022	0.587	0.443
16. Antisocial behavior	40	51.3%	26	49.1%	0.063	0.803	30	73.2%	25	75.8%	0.064	0.800	5.316	0.021	6.008	0.014
17. Antisocial peers	45	57.7%	33	61.1%	0.154	0.694	22	53.7%	23	69.7%	1.974	0.160	0.178	0.673	0.658	0.417
18. Failure in previous interventions	53	69.7%	37	72.5%	0.117	0.732	35	89.7%	27	81.8%	0.939	0.333	5.742	0.017	0.949	0.330
**Family Factors**																
19. Violence between parents	10	12.8%	14	26.4%	3.897	0.048	13	31.7%	13	39.4%	0.474	0.491	6.148	0.013	1.591	0.207
20. Cohabitation problems other than CPV	23	29.5%	29	53.7%	7.838	0.005	21	51.2%	25	75.8%	4.681	0.031	5.447	0.020	4.231	0.040
21. Problematic education style	63	80.8%	43	81.1%	0.003	0.959	37	90.2%	31	93.9%	0.335	0.563	1.798	0.180	2.778	0.096
22. Inversion of the hierarchy	37	48.1%	34	64.2%	3.282	0.070	27	65.9%	26	78.8%	1.505	0.220	3.416	0.065	2.066	0.151
23. Personal problems of parents	15	19.2%	25	46.3%	11.067	0.001	15	37.5%	15	45.5%	0.473	0.492	4.655	0.031	0.006	0.939
24. Non-violent conflicts between parents	27	34.6%	27	50.9%	3.472	0.062	20	48.8%	24	72.7%	4.350	0.037	2.256	0.133	3.999	0.046
**Protective Factors**																
25. Motivation to change	42	55.3%	31	59.6%	0.239	0.625	13	31.7%	19	57.6%	4.985	0.026	5.932	0.015	0.035	0.852
26. Family involvement in therapy	65	84.4%	40	74.1%	2.134	0.144	30	75.0%	23	69.7%	0.256	0.613	1.529	0.216	0.196	0.658
27. Future plans	42	54.5%	29	58.0%	0.147	0.702	9	23.1%	9	27.3%	0.168	0.682	10.406	0.001	7.562	0.006
28. Social support	42	53.8%	35	64.8%	1.579	0.209	18	43.9%	10	30.3%	1.438	0.231	1.063	0.303	9.77	0.002
29. Family support	65	83.3%	40	75.5%	1.226	0.268	25	61.0%	22	68.8%	0.474	0.491	7.289	0.007	0.457	0.499
30. Working alliance in therapy	49	65.3%	35	67.3%	0.053	0.817	21	51.2%	18	54.5%	0.081	0.776	2.207	0.013	1.401	0.237

Note: a, b, c and d are used to explain intragender comparison.

## Data Availability

The data presented in this study are available upon request from the corresponding author. The data are not publicly available due to an ongoing project.
